# Danqi soft capsule prevents infarct border zone remodelling and reduces susceptibility to ventricular arrhythmias in post‐myocardial infarction rats

**DOI:** 10.1111/jcmm.14428

**Published:** 2019-06-24

**Authors:** Shiyu Ma, Jin Ma, Xiaoyi Mai, Xujie Zhao, Liheng Guo, Minzhou Zhang

**Affiliations:** ^1^ The Second Affiliated Hospital of Guangzhou University of Chinese Medicine, Guangdong Provincial Hospital of Chinese Medicine Guangzhou China; ^2^ Guangzhou Key Laboratory of Chinese Medicine for Prevention and Treatment of Myocardial Infarction Guangzhou China

**Keywords:** arrhythmia, cardiac hypertrophy, fibrosis, gap junction, myocardial infarction

## Abstract

Danqi soft capsule (DQ) is a traditional Chinese medicine containing *Salvia miltiorrhiza* and *Panax notoginseng*; it is safe and efficient in treating ischaemic heart diseases. The purpose of the present study was to assess whether DQ could prevent infarct border zone (IBZ) remodelling and decrease ventricular arrhythmias occurrence in post‐myocardial infarction (MI) stage. MI was induced by a ligation of the left anterior descending coronary artery. DQ was administered to the post‐MI rats started from 1 week after MI surgery for 4 weeks. The results showed that DQ treatment significantly attenuated tachyarrhythmia induction rates and arrhythmia score in post‐MI rats. In echocardiography, DQ improved left ventricular (LV) systolic and diastolic function. Histological assessment revealed that DQ significantly reduced fibrotic areas and myocyte areas, and increased connexin (Cx) 43 positive areas in IBZ. Western blot revealed that DQ treatment significantly reduced the protein expression levels of type I and III collagens, α‐smooth muscle actin (α‐SMA), transforming growth factor‐β1 (TGF‐β1) and Smad3 phosphorylation, while increasing Cx43 amounts. Overall, these findings mainly indicated that DQ intervention regulates interstitial fibrosis, Cx43 expression and myocyte hypertrophy by TGF‐β1/Smad3 pathway in IBZ, inhibits LV remodelling and reduces vulnerability to tachyarrhythmias after MI. This study presents a proof of concept for novel antiarrhythmic strategies in preventing IBZ remodelling, modifying the healed arrhythmogenic substrate and thus reducing susceptibility to ventricular arrhythmias in the late post‐MI period.

## 1 INTRODUCTION

Ventricular arrhythmias represent a major cause of morbidity and mortality in post‐myocardial infarction (MI) patients, even in the current era of coronary revascularization, also accounting for a substantial number of sudden cardiac deaths.^1^ Despite the advances in management strategies (eg medical and surgical therapies) and patient education, ventricular arrhythmias represent an unsolved problem, and sudden cardiac death remains an important public health problem.^2^

Increasing evidence reveals that cardiac remodelling at the infarct border zone (IBZ) induced by MI plays a critical role in the occurrence of ventricular arrhythmias.^3^, ^4^ Cardiac remodelling is a progressive process, which starts immediately after acute MI, evolves in the chronic phase of heart failure and finally develops into myocardial hypertrophy, interstitial fibrosis and gap junction remodelling in tissue architecture.^5^ It is closely associated with changes in ventricular size, shape and function after MI.^5^, ^6^ Previous studies have indicated that abnormal conduction exists in the IBZ, constituting the main reason for ventricular tachyarrhythmia (VT) occurrence in MI rats.^3^, ^4^ IBZ remodelling can be considered a primary target for the prevention of ventricular arrhythmias.^7^, ^8^

Traditional Chinese medicine has been applied in the treatment of MI for thousands of years. Danqi soft capsule (DQ) is prepared from a basic formula of two Chinese herbs, including *Salvia miltiorrhiza* and *Panax notoginseng*. The combination of *S miltiorrhiza* and *P notoginseng* is one of the most widely prescribed formulas, with a remarkable protective effect on cardio‐cerebrovascular diseases.^9^, ^10^, ^11^, ^12^ The major effective ingredients of *S Bunge* and *P notoginseng* are salvianolic acids and Panax notoginseng saponins (PNS) respectively.^13^, ^14^ Salvianolic acids prevent cardiac remodelling, including fibrosis and hypertrophy.^15^ Meanwhile, PNS show cardioprotective and antifibrotic effects.^16^, ^17^ Therefore, this study aimed to assess whether DQ prevents IBZ remodelling and decreases VT occurrence after MI.

## 2 METHODS

### 2.1 Ethics statement

All animal procedures were performed in accordance with the National Institutes of Health Guidelines for the Care and Use of Laboratory Animals and the National Standard of the People's Republic of China for Laboratory animal Guidelines for ethical review of animal welfare.

### 2.2 DQ preparation and quality control

The DQ extraction procedure and ultra‐high performance liquid chromatography (UPLC) are described in the supplementary file.

### 2.3 Animal model of myocardial infarction and drug administration

The MI model was carried out as our previously described.^18^ Male Sprague‐Dawley rats (with the weight of 250‐280 g, SPF, Guangdong Medical Experimental Animal Center) were anaesthetized. With the heart exposed by a 1.2 cm lateral thoracotomy, the left coronary artery was permanently ligated 2 mm below the tip of the left auricle with 6‐0 nylon silk. The MI model was considered successfully established when myocardial blanching and cyanosis were visualized within the downstream myocardium. Thorax closure was done with three layers of sutures. The sham‐operated animals (Sham, n = 15) underwent the same procedure except that the silk suture was placed around the left coronary artery without being tied.

One week after MI surgery, all survival rats underwent echocardiography and were randomly grouped into MI group (MI, n = 15), low‐dose DQ group (DQ‐L, n = 15), middle‐dose DQ group (DQ‐M, n = 15) and high‐dose DQ group (DQ‐H, n = 15). DQ rats were orally gavaged with DQ (batch number 14060108; Beijing Changcheng Pharmaceutical Factory, Beijing, China) at a dose of 0.6 g/kg d in the DQ‐L group, 0.9 g/kg d in the DQ‐M group and 1.2 g/kg d in the DQ‐H group started from 1 week after MI surgery for 4 weeks, which was derived from the equivalent conversion between animals and human by body surface area, based on the recommended daily human dosage of DQ.^19^ Other rats were gavaged with an equivalent volume of water. After 4 weeks of treatment, the rats in each group underwent echocardiographic and electrophysiological examinations as described below. Following these procedures, rats were killed and their hearts and blood removed for weighing and other assessments, as also described below.

### 2.4 Echocardiogram

All rats were lightly anaesthetized with 2% sevoflurane. Transthoracic echocardiography was performed using a 21‐MHz phased‐array probe (Vevo 2100, VisualSonics Inc, Canada). Echocardiography was performed in the parasternal short axis B‐ and M‐modes at the papillary muscle level to obtain left ventricular (LV) wall thickness and ejection fraction (EF), and in the parasternal long‐axis view to measure LV end‐systolic and end‐diastolic volumes (LVESV and LVEDV). Each echocardiographic variable was determined in at least four separate LV images acquired from the same heart. The observer was unaware of grouping and treatment.

### 2.5 Programmed electrical stimulation (PES)

Programmed electrical stimulation (PES) in this study was performed essentially as described by Nguyen T^20^ and Nguyen DT.^4^ Induction of ventricular arrhythmias was attempted by ventricular stimulation at a basic cycle length of 120 ms (S1) for eight beats, followed by one to three extra‐stimuli (S2, S3 and S4) at shorter coupling intervals. The end‐point of PES was induction of a VT consisting of at least six consecutive non‐driven ventricular extra‐stimulus beats. A preparation was considered to be non‐inducible when PES produced less than six beats. Distinction was not made between ventricular tachycardia and fibrillation. A VT was considered to be non‐sustained if lasting for 15 beats, and sustained if lasting for >15 beats. A PES‐induced ventricular arrhythmias scoring system was used: 0, non‐inducible preparation; 1, non‐sustained tachyarrhythmias induced with three extra‐stimuli; 2, sustained tachyarrhythmias induced with three extra‐stimuli; 3, non‐sustained tachyarrhythmias induced with two extra‐stimuli; 4, sustained tachyarrhythmias induced with two extra‐stimuli; 5, non‐sustained tachyarrhythmias induced with one extra‐stimulus; 6, sustained tachyarrhythmias induced with one extra‐stimulus; 7, tachyarrhythmias induced during the 20 paced beats at a basic cycle length of 100 ms. If the heart stopped before PES, an arrhythmia score of 8 was assigned.

### 2.6 Histological analysis

Several sections of each heart (5‐µm thick) were prepared, and stained with haematoxylin and eosin for histopathology. Masson's trichrome staining was performed as previously described.^21^ Images were digitized using a digital camera (DP 72, Olympus) under a BX53 microscope (Olympus, Tokyo, Japan). Images were quantified by the CellSens Dimension 1.16 software (Olympus, Tokyo, Japan). The infarct area was expressed as a percentage of the fibrotic scar circumference to the total circumference; the fibrotic area was expressed as a percentage of red‐positive stained area to total tissue area in the IBZ, which was defined a 3‐mm zone adjacent to the infarcted area^22^ using the software.

### 2.7 Immunohistochemical assay

Immunohistochemical staining was performed as described previously.^23^ Heart sections were stained with anti‐connexin (Cx) 43 antibody (1:200, Cell Signaling Technology), with rabbit IgG or serum used instead of primary antibody in negative controls. Peroxidase activity was visualized with the use of diaminobenzidine, and sections were counterstained with haematoxylin. Images were obtained using a DP 72 camera attached to a BX53 microscope (Olympus, Tokyo, Japan). Five fields were selected randomly from each slide for quantitation with the CellSens Dimension software (Olympus, Tokyo, Japan). The percentage of Cx43‐positive staining area represented Cx43 expression.

### 2.8 Quantitative real‐time polymerase chain reaction

After extraction of total ribonucleic acid (RNA) from the mouse hearts using the TRIzol reagent (Invitrogen Life Technologies, USA), RNA was reverse transcribed to produce complementary DNAs (cDNAs) using the Prime Script RT reagent kit (TaKaRa, Japan). cDNA was used as a template for quantitative real‐time polymerase chain reaction (qRT‐PCR) and gene expression was normalized to glyceraldehydes‐3‐phosphate dehydrogenase (GAPDH). The specific forward and reverse primers used were as follows: rats atrial natriuretic peptide (ANP) forward: 5’‐CCTGGACTGGGGAAGTCAAC‐3’ and reverse 5’‐GTCAATCCTACCCCCGAAGC‐3’; rats GAPDH forward: 5’‐ATCCGTTGTGGATCTGACATG‐3’ and reverse 5’‐CAAAGGTGGAAGAATGGGAGT.

### 2.9 Western blot

Protein concentration was determined using a BCA Protein Assay Kit (Novagen, Darmstadt, Germany). Low‐molecular weight marker (Cell Signaling Technology, Beverly, MA) and 50 μg of total protein from samples were separated by 10% SDS‐PAGE. Separated proteins were transferred to a polyvinylidene difluoride membrane, which was blocked at room temperature for 1 hour in Tris‐buffered saline with 0.2% Tween 20 containing 5% skim milk and probed with primary antibodies overnight at 4°C. The diluted concentrations of the primary antibodies (Abcam,�Cambridge,�UK or Cell Signaling Technology, Beverly, MA, USA) were as follows: collagen I (1:500), collagen III (1:500), connexin (Cx) 43 (1:1000), α‐smooth muscle actin (α‐SMA, 1:500), transforming growth factor β1 (TGF‐β1, 1:1000), mothers against decapentaplegic homolog 3 (Smad3, 1:500), phospho‐Smad3 (p‐Smad3, 1:500) and GAPDH (1:1000). Secondary antibodies (Cell Signaling Technology, Beverly, MA) included horseradish peroxidase‐labelled were diluted 1:1000 and incubated for 1h at room temperature. Protein bands on Western blots were visualized with ECL Plus (Millipore, Billerica, MA). After immunoblotting, the film was scanned and the intensity of immunoblot bands was detected with the ImageJ (NIH imaging) analysis software. Relative protein band intensities were based on GAPDH signals. The final results were expressed as fold changes by normalization to control values.

### 2.10 Enzyme‐linked immunosorbent assay

Blood was taken from the abdominal aorta. The serum was separated by centrifugation and immediately stored at −80°C until further analysis. The serum levels of brain natriuretic peptide (BNP), monocyte chemoattractant protein (MCP)‐1 and TGF‐β1 were determined by ELISA using commercially available kits (RD, RD Systems, Minneapolis, USA), according to the manufacturer's instructions. Absorbance was measured at 450 nm with background reading at 570 nm on a microplate reader. The concentration of each sample was calculated according to the standard curve. All concentrations were expressed in pg/mL.

### 2.11 Data analysis and statistics

Data are mean ± standard deviation. All differences were normally distributed variables except arrhythmia scores and were examined by one‐way anova; post hoc analyses were performed by Bonferroni correction for multiple comparison. Arrhythmia scores were examined by Kruskal‐Wallis anova, with post hoc Dunn's test. The SPSS statistical software (SPSS, IL) was used for statistical analyses, and *P* < 0.05 indicated statistical significance.

## 3 RESULTS

### 3.1 DQ improves cardiac function after MI

**Figure 1:**
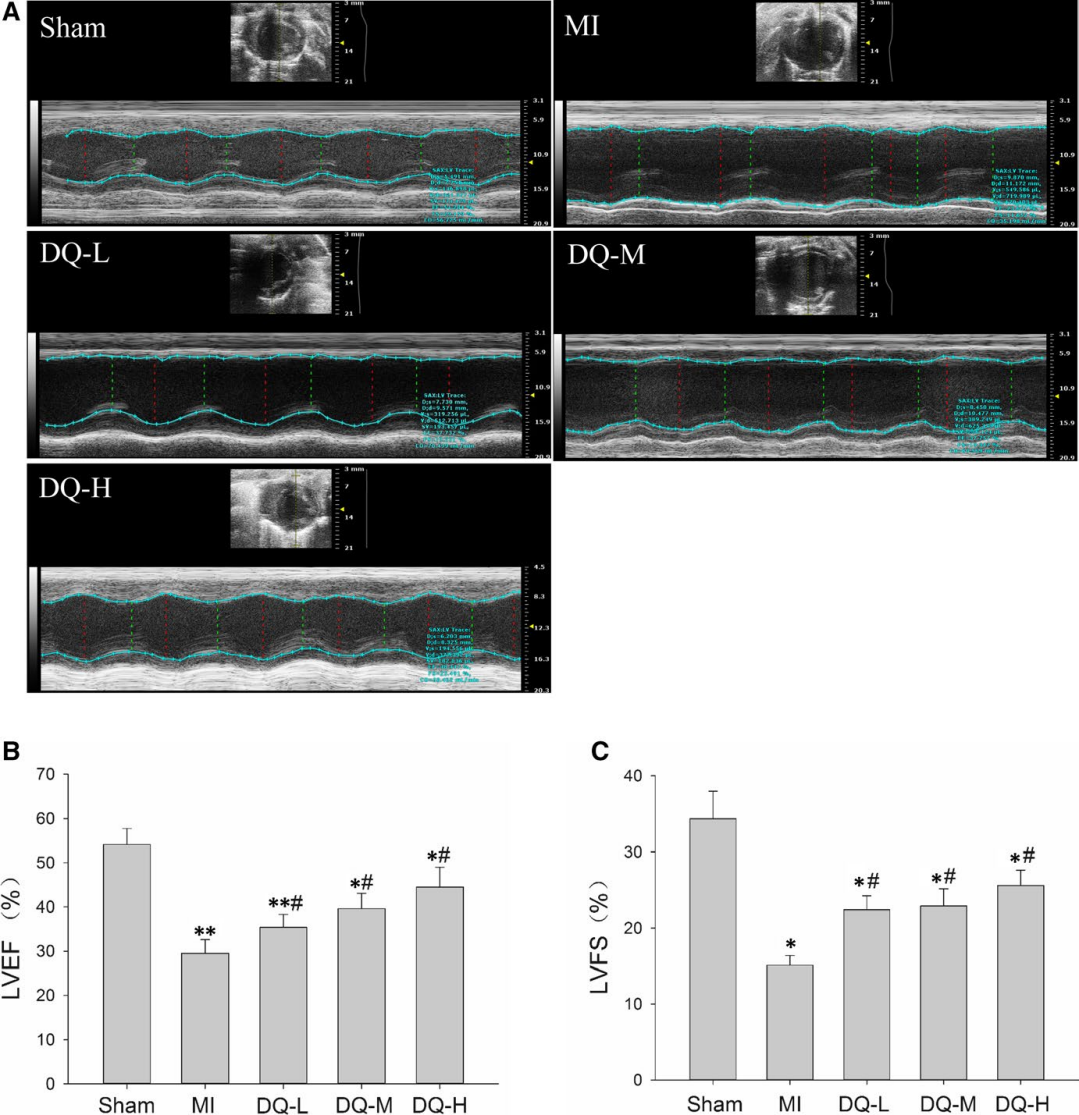
Effects of DQ on cardiac structure and function. A, Representative examples of M‐mode echocardiograms of the three groups 5 weeks after MI. B, LVEF. C, LVFS. D, LVIDd. E, LVIDs. *P < 0.05, **P < 0.01 vs Sham group, ^#^ *P* < 0.05 vs MI group, n = 15 rats/group

Five weeks after MI modelling, echocardiography showed clear anterior wall motion abnormality (Figure 1A), and ventricular systolic dysfunction was significantly reduced in the MI group (Figure 1B,C). EF in MI rats decreased from 54.1 ± 3.7% at baseline to 29.5 ± 3.1% compared with the Sham group *(P* < 0.01, Figure 1B). Left ventricle fraction shortening in MI rats decreased from 34.4 ± 3.6% to 15.1 ± 1.3% compared with the Sham group *(P* < 0.05, Figure 1C). However, rats administered DQ at low, middle and high doses, respectively, showed significantly increased EF (*P* < 0.05, Figure 1B) and FS (*P* < 0.05, Figure 1C) compared with the MI group. Both diastolic LV internal dimension (LVIDd, *P* < 0.01, Figure 1D) and end‐systolic LV internal dimension (LVIDs, *P* < 0.01, Figure 1E) were elevated in the MI group, but decreased after DQ treatment (both *P* < 0.05).

### 3.2 DQ inhibits susceptibility to VT in rats with MI

**Figure 2:**
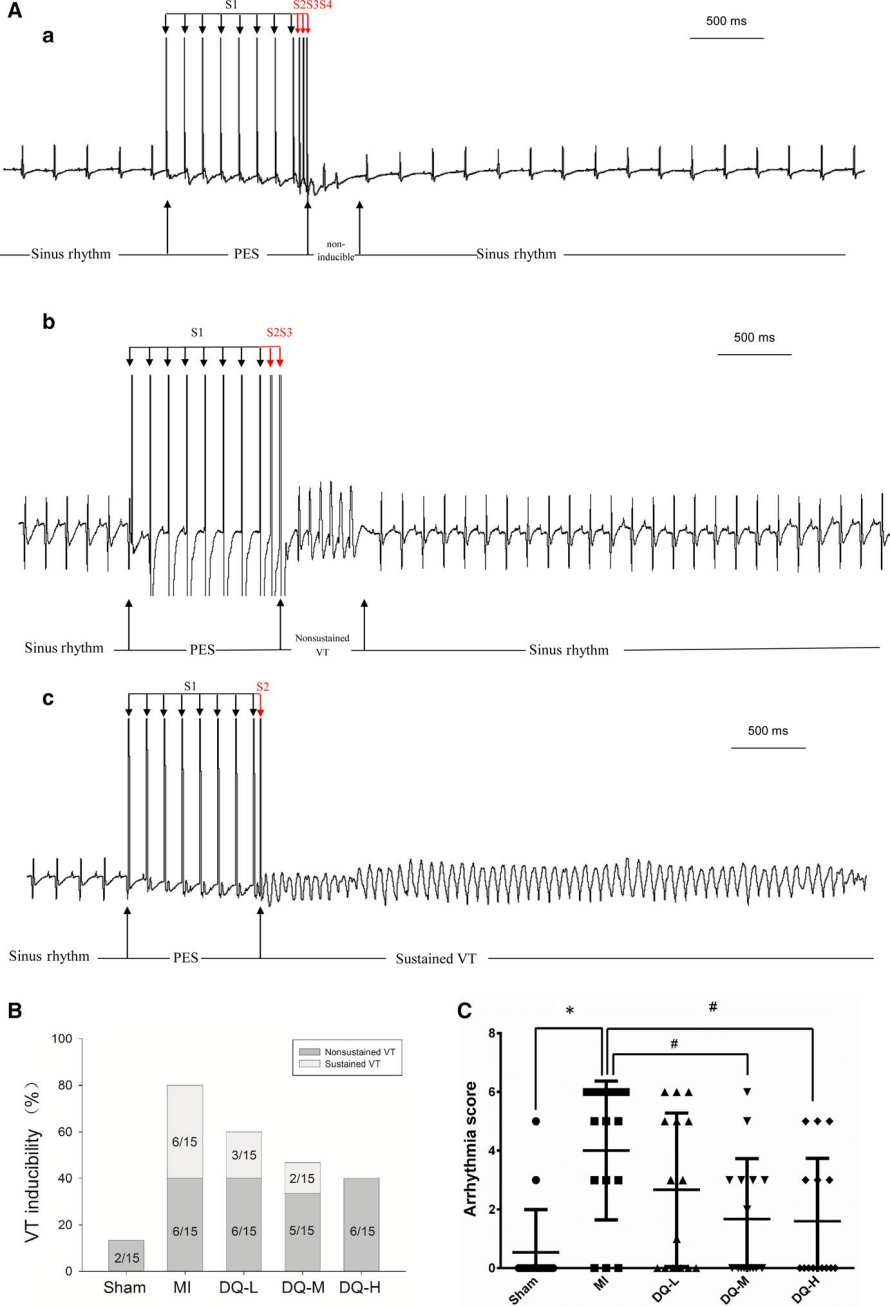
Effects of DQ on PES‐induced ventricular tachyarrhythmia. A, Various ventricular arrhythmias induced by programmed electrical stimulation in rats. a, Eight basic stimuli (S1) at cycle lengths of 120 ms and three extra‐stimuli (S2, S3 and S4) induced no ventricular tachycardia (VT). b, Eight basic stimuli (S1) and two extra‐stimuli (S2 and S3) induced non‐sustained VT. c, Eight basic stimuli (S1) and one extra‐stimulus (S2) induced sustained VT. B, Inducibility of VT 5 weeks after infarction. C, Scores of inducible VT. *P < 0.05 vs Sham group, ^#^ *P* < 0.05 vs MI group, n = 15 rats/group

Figure 2A shows typical examples of no arrhythmia (a), non‐sustained VT (b) and sustained VT (c) induced by programmed stimulation in rats. The rates of VT induction were 80% (12/15, including six sustained and six unstained VT) in the MI group, and 13.3% (2/15, both unstained VT) in the Sham group. VT induction rates in the DQ groups were significantly decreased (9/15, 60% in the DQ‐L group; 7/15, 46.7% in the DQ‐M group; 6/15, 40% in the DQ‐L group; Figure 2B). Arrhythmia scores in the DQ‐M and DQ‐L groups were also lower than those of the MI group (*P* < 0.05, Figure 2C). The conduction velocity was lower in the MI group than in the Sham group (*P* < 0.01, Supporting Information). This decrease was reversed after administering DQ, with a recovery of conduction velocity observed in DQ‐treated groups. These results suggested that DQ could reduce susceptibility to PES VT and increase conduction velocity after MI.

### 3.3 DQ inhibits cardiac fibrosis in IBZ

**Figure 3:**
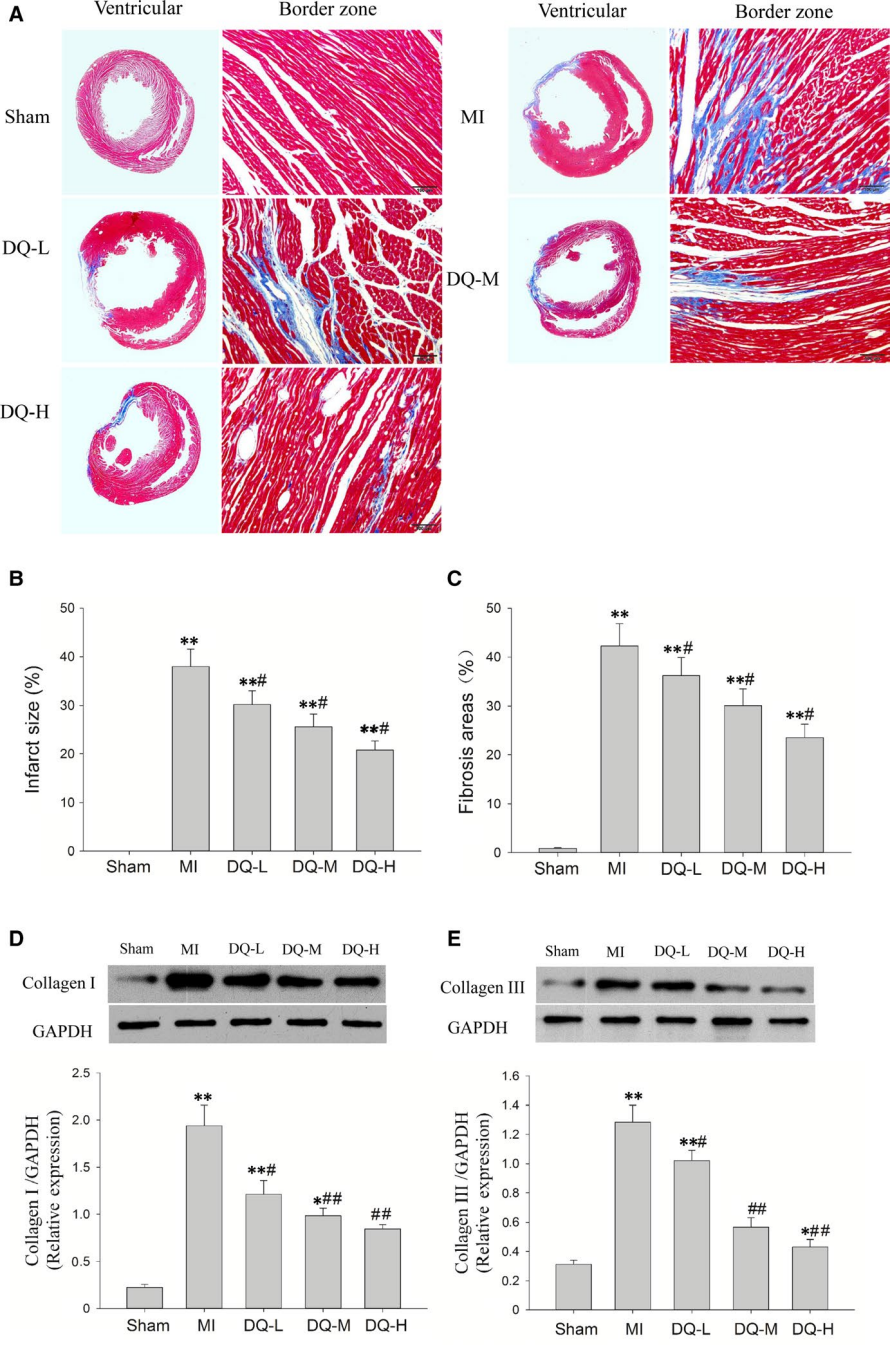
Effect of DQ on interstitial fibrosis. A, Representative images showing ventricular myocardial fibrosis (Masson stain, which stains fibrosis blue and viable muscle red). B, Statistical results of percentage of infarcted areas (n = 5 rats/group). C, Statistical results of fibrosis areas in the infarct border zone. DQ reduced fibrosis‐positive area (n = 5 rats/group). D, DQ reduced the protein levels of collagen Ⅰ as assessed by Western blot (n = 5 rats/group). E, DQ reduced the protein levels of collagen III as assessed by Western blot (n = 5 rats/group). *P < 0.05, **P < 0.01 vs Sham group, ^#^ *P* < 0.05, ^##^ *P* < 0.01 vs MI group

Histology confirmed large infarcted areas (Figure 3A), with an infarct percentage of 38% ± 3.6% in MI rats (Figure 3B); the fibrotic tissue within the IBZ accounted for 42.3 ± 4.5% of total area (Figure 3C). DQ effectively decreased the total infarct areas (*P* < 0.05, Figure 3B) and fibrotic areas in the IBZ (*P* < 0.05, Figure 3C). To quantify the impact of DQ on collagen amounts, type I and III collagen were detected by Western blot. MI resulted in significantly increased type I and III collagen deposition (Figure 3D and Figure 3E). DQ treatment markedly reduced type I (*P* < 0.05 in the DQ‐L group, and *P* < 0.01 in the DQ‐M and DQ‐H groups; Figure 3D) and III (*P* < 0.05 in the DQ‐L group, and *P* < 0.01 in the DQ‐M and DQ‐H groups; Figure 3E) collagen deposition.

### 3.4 DQ reverses Cx43 expression and distribution in the IBZ

**Figure 4:**
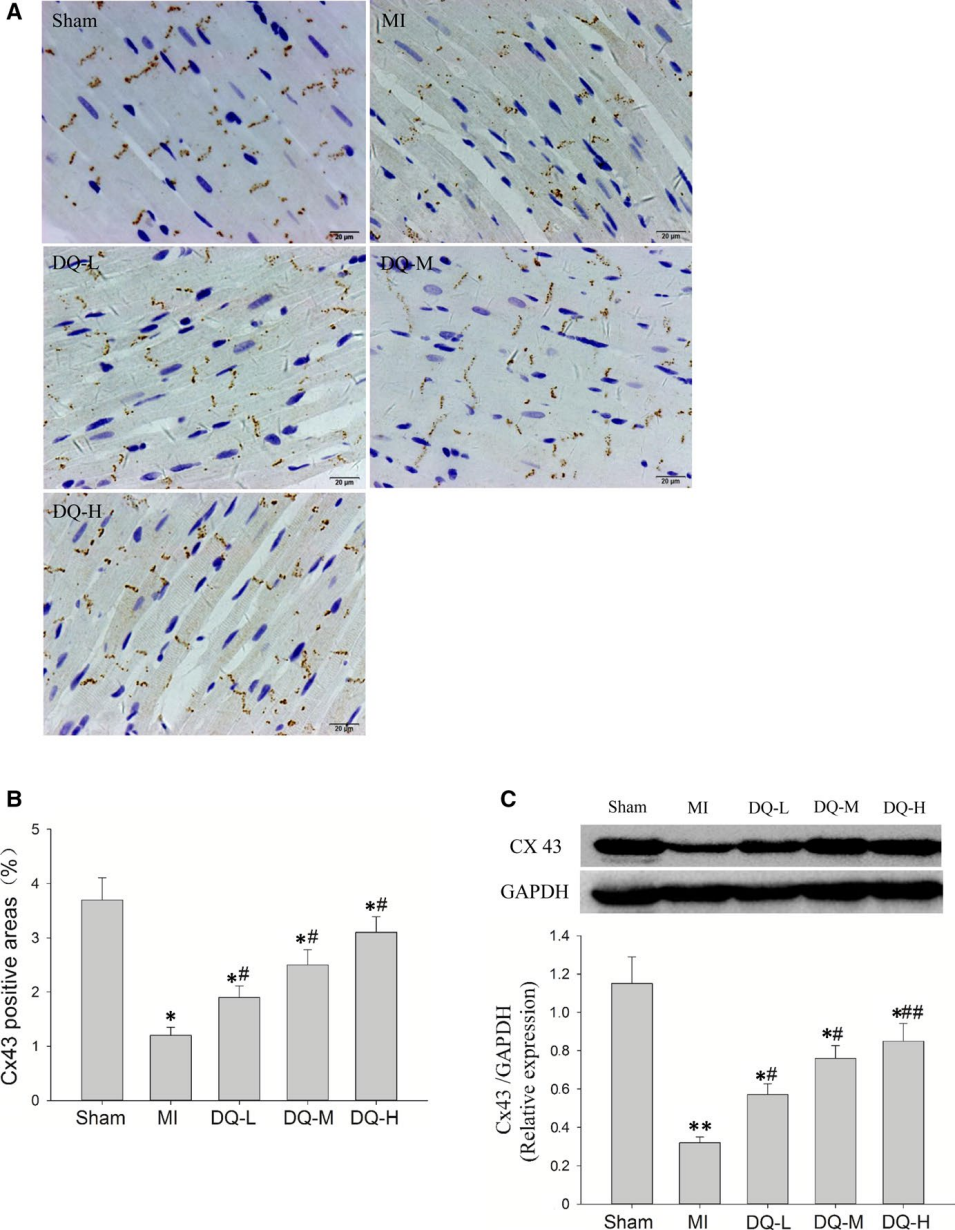
Effect of DQ on Cx43 expression and distribution. A, Representative immunohistochemical staining data for the infarct border zone (Scale bar: 20 um). B, Percentages of Cx43 positive areas among the four groups (n = 5 rats/group). C, TQ increased the protein levels of Cx43 in the infarct border zone as assessed by Western blot (n = 5 rats/group). *P < 0.05, **P < 0.01 vs Sham group, ^#^ *P* < 0.05, ^##^ *P* < 0.01 vs MI group

Immunohistochemistry showed the Cx43 staining in LV sections of sham‐operated rats, predominantly localized to the myocyte‐myocyte junction corresponding to intercalated discs. Cx43 in the MI model group was clearly decreased and had a disordered and uneven distribution (Figure 4A). The immunohistochemical levels of Cx43 were significantly reduced in the IBZ in MI rats compared with Sham animals (*P* < 0.05, Figure 4B). Meanwhile, Western blot showed that Cx43 protein levels in the IBZ were markedly decreased in MI rats compared with Sham animals (*P* < 0.01, Figure 4C). The expression and distribution of Cx43 in the DQ group were significantly reversed compared with the MI group (*P* < 0.05 in the DQ‐L and DQ‐M groups; *P* < 0.01 in the DQ‐H group).

### 3.5 DQ inhibits myocyte hypertrophy in the IBZ

**Figure 5:**
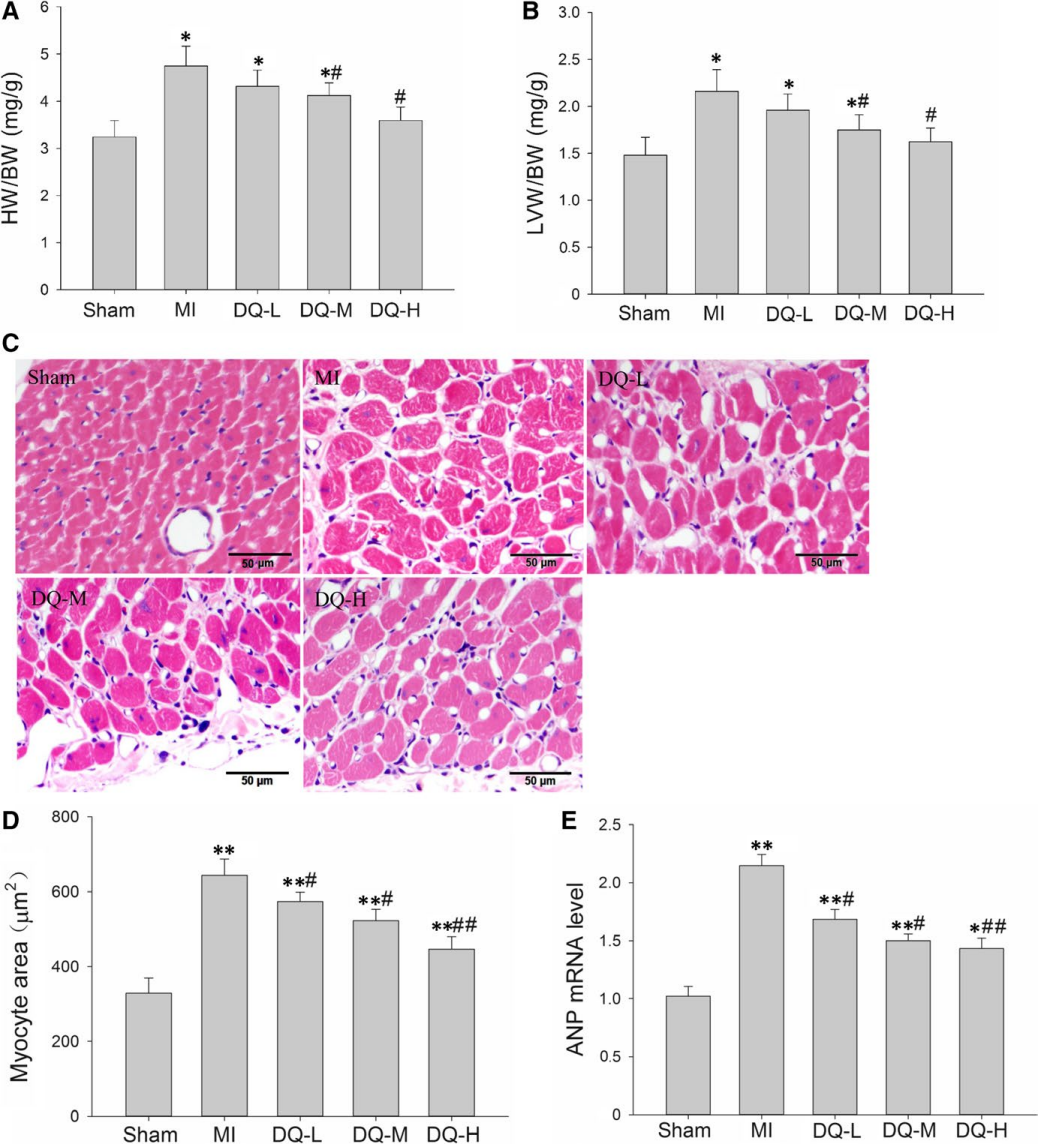
Effect of DQ on myocyte hypertrophy. A, Quantitative analysis of HW/BW (n = 10 rats/group). B, Quantitative analysis of LVW/BW (n = 10 rats/group). C, Haematoxylin and eosin staining of representative cross‐sections of cardiac myocytes. D, Quantitative analysis of myocyte cross‐sectional areas (n = 5 rats/group). E, ANP mRNA qRT‐PCR analysis (n = 5 rats/group). *P < 0.05, **P < 0.01 vs Sham group, ^#^ *P* < 0.05, ^##^ *P* < 0.01 vs MI group

Five weeks post‐MI, rats developed significantly increased heart weight (HW)/bodyweight (BW) ratios (*P* < 0.05, Figure 5A) and LV weight (LVW)/HW ratios (*P* < 0.05, Figure 5B); meanwhile, DQ inhibited the above indexes (*P* < 0.05 in the DQ‐M and DQ‐L groups). Haematoxylin and eosin staining was used to assess myocyte morphology and transverse diameters (Figure 5C). Five weeks post‐MI, animals developed a higher degree of hypertrophy with a sparser myocyte distribution and increasing myocyte area; DQ treatment inhibited MI‐induced myocyte hypertrophy and showed a denser myocyte distribution and reduced myocyte area (Figure 5D). Results of the qRT‐PCR also showed that the enhanced expression levels of the hypertrophic markers natriuretic peptide ANP were significantly decreased in DQ rats in comparison to MI rats (Figure 5E). Collectively, our results suggest that DQ inhibited myocyte hypertrophy in IBZ.

### 3.6 DQ inhibits myofibroblast differentiation in MI rats and TGF‐β1/Smad3 pathway

**Figure 6:**
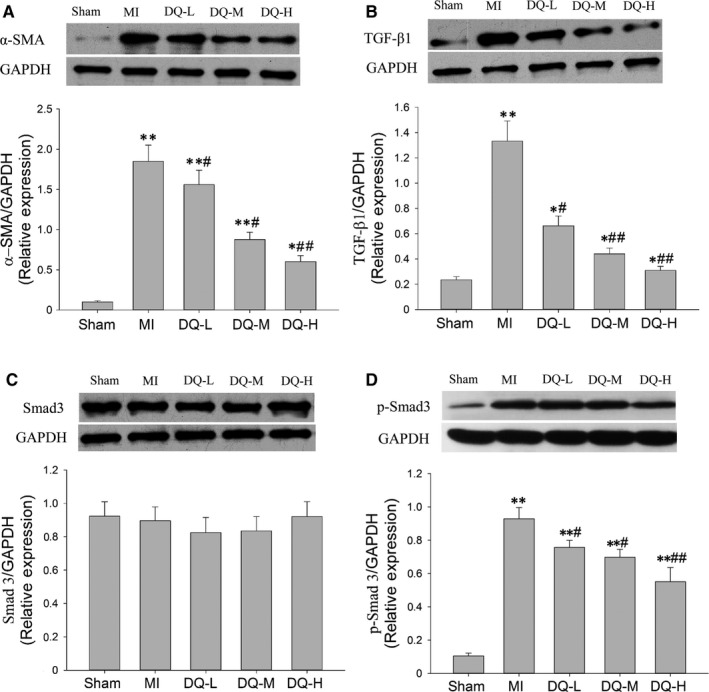
Effect of DQ on fibroblasts differentiation to myofibroblasts. A, DQ reduced the protein levels of α‐SMA as assessed by Western blot (n = 5 rats/group). B, DQ reduced the protein levels of TGF‐β1 as assessed by Western blot (n = 5 rats/group). C, DQ had no effect on the protein levels of Smad3 as assessed by Western blot (n = 5 rats/group). D, DQ reduced the protein levels of p‐Smad3 as assessed by Western blot (n = 5 rats/group). *P < 0.05, **P < 0.01 vs Sham group, ^#^ *P* < 0.05, ^##^ *P* < 0.01 vs MI group

In MI rats, α‐SMA was markedly increased in the IBZ compared with Sham rats (*P* < 0.01, Figure 6A). DQ treatment resulted in reduced protein expression of α‐SMA compared with MI rats (*P* < 0.05 in the DQ‐L and DQ‐M groups, and *P* < 0.01 in the DQ‐H group; Figure 6A). TGF‐β1 levels were markedly increased in the MI group compared with the Sham group (*P* < 0.01, Figure 6B). DQ attenuated the TGF‐β1 level increase after MI (*P* < 0.05 in the DQ‐L group, and *P* < 0.01 in the DQ‐M and DQ‐H groups; Figure 6B). Smad3 amounts showed no difference among the five groups *(P* > 0.05, Figure 6C). But DQ decreased Smad3 phosphorylation (p‐Smad3) after MI (*P* < 0.05 in the DQ‐L group, and *P* < 0.01 in the DQ‐M and DQ‐H groups; Figure 6D).

### 3.7 DQ decreases the serum level of BNP, MCP‐1 and TGF‐β1

**Figure 7:**
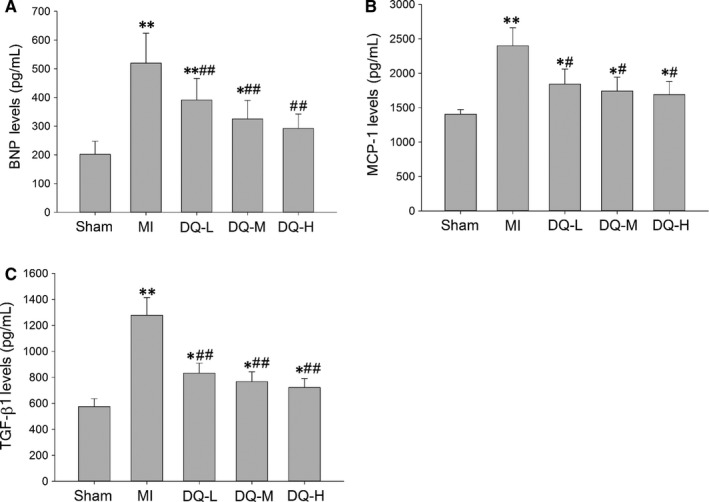
Effects of DQ on serum levels of BNP, MCP‐1 and TGF‐β1. A, Serum levels of BNP, decreased by DQ (n = 10 rats/group). B, Serum levels of MCP‐1, decreased by DQ (n = 10 rats/group). C, Serum levels of TGF‐β1, decreased by DQ (n = 10 rats/group). *P < 0.05, **P < 0.01 vs Sham group, ^#^ *P* < 0.05, ^##^ *P* < 0.01 vs MI group

As shown in Figure 7, serum levels of BNP, MCP‐1 and TGF‐β1 were all increased in the MI group compared with Sham animals (*P* < 0.01). After 4 weeks of DQ treatment the increased levels of BNP, MCP‐1 and TGF‐β1 were reversed in the three different dose groups compared with the MI group (BNP, *P* < 0.01, Figure 7A; MCP‐1, *P* < 0.05, Figure 7B; TGF‐β1, *P* < 0.01, Figure 7C).

## 4 DISCUSSION

The Chinese herb combination *S miltiorrhiza* and *P notoginseng* is one of the most widely prescribed formulas, and has remarkable protective effects on heart failure^10^ and coronary heart disease.^24^, ^25^ The present study demonstrated the considerable long‐term cardiac protective effects of this herb combination (DQ) in IBZ remodelling, decreasing susceptibility to VT in post‐MI rats. The current results showed that DQ inhibited fibrosis and myocyte hypertrophy, and increased Cx43 expression in the IBZ. DQ also inhibited fibroblasts differentiation into myofibroblasts and decreased the serum levels of BNP, MCP‐1 and TGF‐β1.

Ventricular arrhythmia episodes are responsible for most sudden cardiac deaths, with MI being the principal underlying cause.^26^ Ventricular arrhythmias in patients or animal models with MI are thought to be associated with structural remodelling within the IBZ.^4^, ^27^, ^28^ Human scar tissue heterogeneity in the IBZ, measured by late gadolinium enhancement magnetic resonance imaging (LGE MRI) has recently been shown to predict ventricular arrhythmias risk and improve risk stratification in patients with chronic MI.^28^ During the MI healing phase, dead cardiomyocytes are slowly replaced by collagen, leading to the formation of a scar with a dense collagenous core surrounded by a thin layer of surviving myocardium, known as the IBZ.^26^ IBZ remodelling creates the substrate for re‐entrant circuits and conduction dysfunction causing late post‐MI VT.^3^, ^29^ Therapeutic strategies to homogenize infarct scarring, either by ablation or pharmacologically, are antiarrhythmic in the chronically infarcted heart.^30^, ^31^ In this study, more interstitial fibrosis, myocyte hypertrophy and lower expression of Cx 43 occurred in the IBZ 5 weeks after MI compared with Sham rats, which could provide a vulnerable substrate for VT. Treatment with DQ at low, middle and high doses 1 week after MI for 4 weeks effectively inhibited cardiac remodelling in the IBZ and decreased VT inducibility as well as VT scores in rats. Previous studies also reported similar conclusions that pirfenidone,^20^, ^32^ relaxin^8^ and GS‐6201^33^ 1 week after MI for 4 weeks decrease IBZ remodelling and reduce susceptibility to VT in MI rats. Drug targeted at IBZ remodelling may be an effective treatment for post‐MI VT.

Interstitial fibrosis is the main pathological change in post‐MI structural remodelling in the IBZ.^34^ Excessive collagen, generated by activated fibroblasts, increases the stiffness of the myocardial wall and is linked to cardiac remodelling, thereby impeding normal electrical conduction function.^35^ Diffuse interstitial fibrosis is an independent predictor of the likelihood of developing electrophysiological disturbances resulting in cardiac arrhythmia.^36^ Therefore, inhibiting or reversing fibrosis is an established target of many interventions for treating ventricular arrhythmias.^37^ These interventions may lead to a partial recovery of conduction function in the IBZ by decreasing fibrosis, thereby reducing susceptibility to VT.^4^ In the present study, DQ markedly decreased LV total infarct scar and IBZ fibrotic areas compared with the Sham group. DQ is a Chinese medicinal herb containing multiple compounds as assessed by UPLC‐MS (results in supplementary file). Tanshinol, salvianolic acid B, tanshinone IIA, cryptotanshinone, ginsenoside Rg1, ginsenoside Rb1 and ginsenoside R1 in rat plasma were detected after oral administration of DQ.^38^ The half‐lives of the above components are 2.23 h, 4.02 h, 4.61 h, 1.95 h, 5.38 h, 187.57 h and 4.95 h.^38^ Salvianolic acid B,^39^ Ginsenoside Rg1,^40^ Ginsenoside Rb1^41^ and tanshinone IIA^42^ have antifibrotic effects demonstrated in different organ fibrosis models, including cardiac fibrosis, which explains the antifibrotic effect of DQ. Fibroblasts differentiation into myofibroblasts plays a key role in cardiac fibrosis.^43^ In the present study, DQ reduced α‐SMA and type I and III collagen expression levels in MI rats, indicating that it could inhibit cardiac fibroblast‐myofibroblast transition in vivo. TGF‐β1 is a major mediator of myofibroblast formation after MI.^44^ TGF‐β1 and p‐Smad3 were up‐regulated in the MI group and reversed after treatment with DQ for 4 weeks. The serum levels of BNP, MCP‐1 and TGF‐β1 were also decreased in the DQ group compared with the MI group. These results indicate that DQ inhibits myofibroblast transition by inhibiting the TGF‐β1/Smad3 pathway or some related factors. DQ decreased susceptibility to VT by inhibiting IBZ fibrosis through its inhibitory effect on myofibroblast transition.

Traditionally, interstitial fibrosis is considered to alter cardiac electrophysiology by mechanically separating myocytes and creating barriers to propagation of the electrical impulse. There is increasing evidence that fibroblasts may also actively contribute to cardiac electrophysiology and arrhythmogenesis through direct electrical coupling.^45^ Gap junction abnormality is as a key molecular feature of the arrhythmogenic substrate.^46^ Theoretical modelling and studies of connexin expression in diseased human and experimental animal hearts suggest that altered gap junction protein expression or function may underlie the high incidence of arrhythmias associated with many forms of heart disease, including hypertrophic, ischaemic and dilated cardiomyopathy.^47^, ^48^, ^49^, ^50^ Cx43 is the most abundant and arguably the most important physiologically relevant protein in the working myocardium of the ventricle. It was reported that cardiac‐restricted knockout of Cx43 causes a conduction disorder and sudden cardiac death from spontaneous ventricular arrhythmias in mice.^46^ Low levels of Cx43 in the gap junction have been reported in the hypertrophied and ischaemic human heart, and depleting gap junction plaques containing Cx43 slow ventricular conduction velocity, leading to arrhythmia and sudden cardiac death.^48^, ^49^, ^51^ In the present study, MI resulted in decreased Cx43 expression in the IBZ, corroborating previous reports on Cx43 level changes in experimental MI models.^52^, ^53^ Further, DQ effectively increased Cx43 levels in the IBZ 5 weeks after MI, thereby reducing vulnerability/susceptibility to VT, by up‐regulating Cx43 in the IBZ.

Cardiomyocytes are post‐mitotic cells, and cannot be regenerated in the heart after cell death. Evidence suggests that cardiac cells, both cardiomyocytes and interstitial cells, are continually replaced by newer cell populations. The characteristic histopathological pattern after MI represented by cardiomyocyte hypertrophy and cellular disorganization, coupled with fibrosis, predisposes victims to the onset of ventricular arrhythmias, which are reflected by abnormal electrophysiological conduction and the electric instability of the myocardium.^54^, ^55^, ^56^ Myocardial hypertrophy alters longitudinal and transverse conduction rates, and elevated non‐uniformity of conduction velocity anisotropy induces changes of intracellular conductance, which can create conditions required to generate re‐entrant arrhythmias.^57^, ^58^ TGF‐β1 plays a pivotal role in the development of cardiac hypertrophy.^59^ This study found that MI resulted in myocyte hypertrophy in the IBZ. After 4 weeks of treatment, DQ decreased the myocyte diameter and the protein expression of TGF‐β1 and p‐Smad3, which indicated DQ‐inhibited myocyte hypertrophy by TGF‐β1/Smad3 pathway. Therefore, DQ may reduce susceptibility to VT after MI by inhibiting myocyte hypertrophy in the IBZ.

## 5 CONCLUSION

In summary, the present study mainly demonstrated that DQ reduces late susceptibility to PES‐induced VTs in the chronic healed infarct phase by inhibiting remodelling in the IBZ, including regulating interstitial fibrosis, Cx43 expression and myocyte hypertrophy. The principal and important finding of this study is the proof of concept of a novel antiarrhythmic strategy for modifying infarct healing by drugs, which modifies the healed arrhythmogenic substrate by inhibiting remodelling at the healed IBZ, thus reducing VT inducibility in the late post‐MI period.

## CONFLICT OF INTEREST

The authors declare no conflict of interest.

## AUTHOR CONTRIBUTIONS

Shiyu Ma and Jin Ma conceived the study, designed, performed and analysed the experiments, carried out the data collection and wrote the paper. Xiaoyi Mai, Xuejie Zhao and Liheng Guo carried out the data collection. Minzhou Zhang coordinated the study and revised the paper. All authors reviewed the results and approved the final version of the manuscript.
