# Supplementary material for: Danqi soft capsule prevents infarct border zone remodelling and reduces susceptibility to ventricular arrhythmias in post‐myocardial infarction rats
Source: J Cell Mol Med. 2019 Jun 24;23(8):5454–65. doi: 10.1111/jcmm.14428 (PMC6653321; doi:10.1111/jcmm.14428)
Supplement: Supplementary file 1 [file JCMM-23-5454-s001.doc]

**Danqi soft capsule prevents infarct border zone remodeling and reduces susceptibility to ventricular arrhythmias in post-myocardial infarction rats**

Shiyu Ma^1, 2^, Jin Ma^1^, Xiaoyi Mai^1, 2^, Xujie Zhao^1^, Liheng Guo^1^, Minzhou Zhang^1, 2, *^

**Supplementary file**

**Methods**

**Preparation of Danqi soft capsule (DQ)**

DQ consist of two traditional Chinese herbs as shown in Table S1. All herbs were selected from genuine regional herbs by China Meheco Great Wall Pharmaceutical Co. Ltd (Beijing, China), and identified by the thin layer chromatography (TLC) method referring to Appendix VI B of Chinese Pharmacopoeia 2005 Edition. The criteria of DQ pharmaceutical preparation (YBZ00332005-2007Z) were issued by China Food and Drug Administration. Briefly, after drying, *Salvia miltiorrhiza* (200g) was macerated at room temperature with distilled water for 8 times (v/w), and decocted thrice for 1h each. The filtrates were mixed and condensed at -0.09 Mpa and 50^o^C to a final concentration of 1.35~1.4 g/ml (weight of original herb/volume of solution). *Panax notoginseng* was ground into fine powder and mixed with the concentrate of *Salvia miltiorrhiza*. The mixture was dried and pulverized again. The excipients were plant oil matrix mixed with heated soybean oil, beeswax and span-80 at the ratio of 18:1:1. The excipients and herb mixture powder were mixed at the ratio of 1:1:1.8 and produce 1000 soft capsules.

**Ultra-high performance liquid chromatography (UPLC) fingerprint and identification of the constituents of DQ**

A fingerprint method for analyzing DQ by UPLC (ACQUITY UPLCTM, Waters Corp, USA) was established, and combined UPLC/Q-TOF-MS/MS (Bruker Daltonics, Bremen, Germany) was used for the identification of constituents. Briefly, the analysis of 80% methanolic extract of DQ was performed on a BEH C18 column (2.1 mm×50 mm, 1.7μm), with 0.1% formic acid aqueous solution (A) and 1% formic acid acetonitrile (B) as mobile phase using the following elution gradient: 5% B ( 0-3 min), 18%~23% B ( 3~13 min), 23%~40% B (13~15 min), 40%~70% B (18~18 min), and 70%~100% B (18~23 min). The flow rate was 250μL/min, with detection wavelength set at 208 nm and column temperature maintained at 30℃. An aliquot of 2µL of the test solution was injected into the UHPLC system. Then, quadrupole-time of flight-mass spectrometry (Q-TOF-MS) was used for qualitative analysis under positive and negative ion modes.

**Multielectrode array measurement**

For multielectrode array (MEA) mapping, the epicardial surface rested on the MEA culture dish (Multi Channel Systems, Reutlingen, Germany) and continuously superfused with oxygenated modified Tyrode solution with at 37 °C. The electrode arrays were mounted onto a printed circuit board and then fitted into the System interface. Electrical stimulation (bipolar pulses, 1–7 V, 1000-µs duration, 5 Hz frequency) was applied via one of the MEA microelectrodes. Data were sampled at 10 kHz per channel with simultaneous data acquisition using the Cardio 2D software, and five fields were recorded. All the data were analyzed to generate activation maps and measure conduction velocity (CV) using the Cardio 2D+ software (Multi Channel Systems).

**Results**

**Chemical constituents of DQ**

Retention times, UV absorption data, and a chemical library were combined to determine the chemical composition of DQ; 25 chemical components in the chromatogram of DQ were resolved (Figure S1, Table S2).

**DQ improves conduction function in rats with post-MI**

The isochronal map demonstrated a typical large conduction block zone in the infarct border zone (IBZ), which could block wave propagation in the myocardial infarction (MI) group (Fig. S2A). The activation, which was located distally, propagated to the block zone. The CV was lower in the MI group than in the sham group (*P* < 0.01, Fig. S2B). This decrease was reversed after administering DQ, with a recovery of CV observed in DQ-treated groups. These results indicated that DQ improved IBZ conduction function after MI.

**TABLE S1. The information of Chinese medicines in DQ.**

| **Chinese**  **name** | **Latin name** | **Family** | **Place of Origin (Province)** | **Used part** | **Major compound in modern pharmacology study** |
| --- | --- | --- | --- | --- | --- |
| Sanqi | Panax notoginseng | Araliaceae | Yunnan | rhizome | Notoginsenoside R1, Ginsenoside Rg1 |
| Danshen | Salvia miltiorrhiza | Labiatae | Jiangsu | rhizome | Salvianic acid A,  Salvianolic acid B |

Note: Panax notoginseng and Salvia miltiorrhiza dosage ratio is 1:1.

**
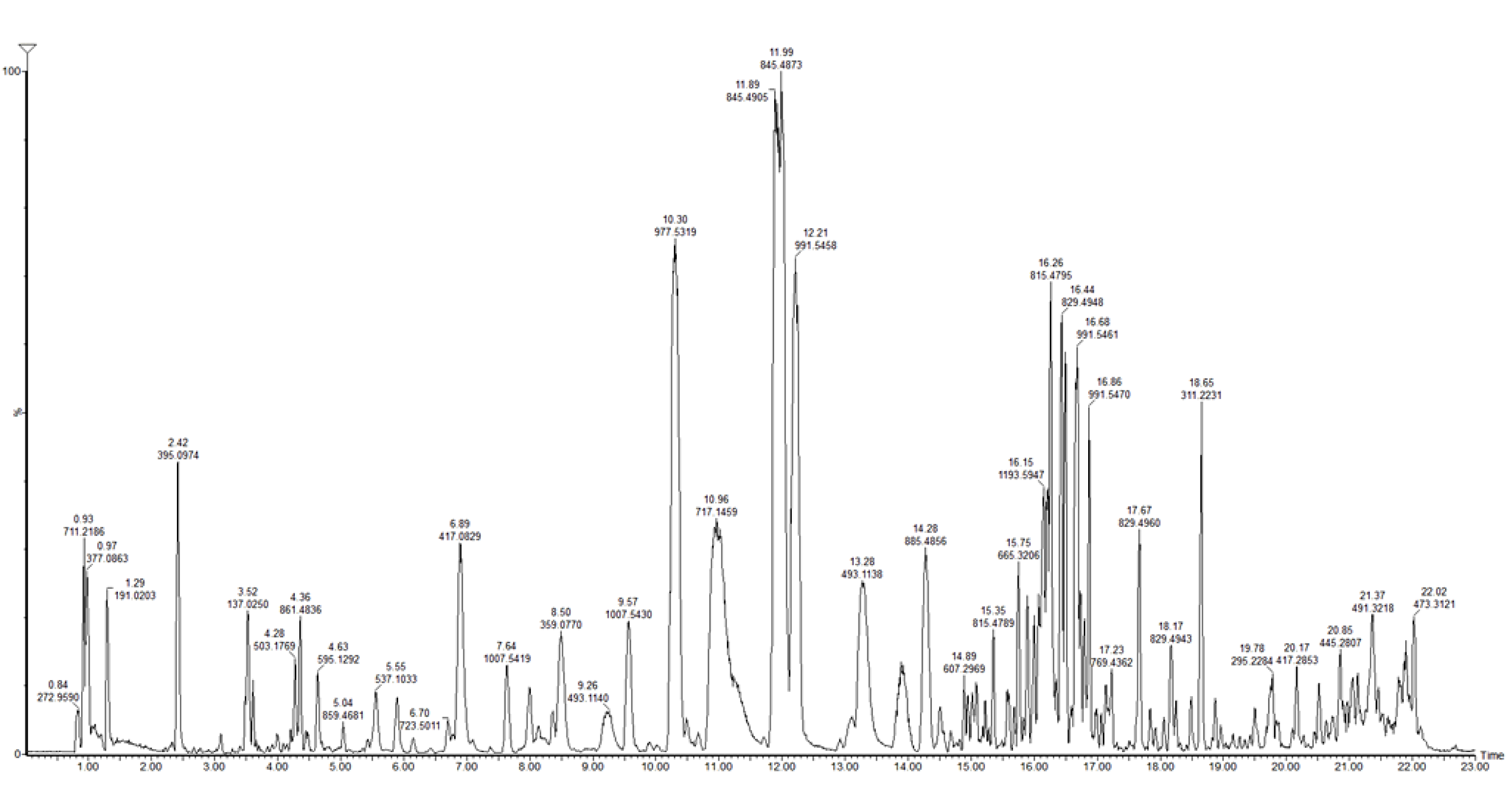
**

**FIGURE S1.** Chromatograms of the isolated DQ compounds.

**TABLE S2.** Chemical constituents of DQ

| ***No.*** | **R*t* / min** | **Compound** | **Element composition** | **Source** |
| --- | --- | --- | --- | --- |
| 1 | 2.44 | Salvianic acid A | C9H10O5 | Salvia miltiorrhiza |
| 2 | 4.36 | Notoginsenoside H | C48H82O21 | Panax notoginseng |
| 3 | 4.67 | 10-Hydroxy-4,6-decadiynoic acid | C22H32O13 | Panax notoginseng |
| 4 | 5.55 | Lithospermic acid | C27H22O12 | Salvia miltiorrhiza |
| 5 | 7.64 | Notoginsenoside R3/Notoginsenoside R6 | C48H82O19 | Panax notoginseng |
| 6 | 9.57 | Notoginsenoside M/Notoginsenoside N | C48H82O19 | Panax notoginseng |
| 7 | 10.26 | Salvianolic acid B | C36H30O16 | Salvia miltiorrhiza |
| 8 | 10.30 | Notoginsenoside R1 | C47H80O18 | Panax notoginseng |
| 9 | 10.96 | Lithospermic acid B | C36H30O16 | Salvia miltiorrhiza |
| 10 | 11.99 | Ginsenoside Rg1 | C42H72O14 | Panax notoginseng |
| 11 | 12.21 | Ginsenoside Re | C48H82O18 | Panax notoginseng |
| 12 | 13.28 | Salvianolic acid A | C26H22O10 | Salvia miltiorrhiza |
| 13 | 15.35 | Notoginsenoside R2/Ginsenoside F5 | C41H70O13 | Panax notoginseng |
| 14 | 15.58 | Notoginsenoside M/Notoginsenoside N | C48H82O19 | Panax notoginseng |
| 15 | 16.01 | GinsenosideRf | C42H72O14 | Panax notoginseng |
| 16 | 16.06 | Ginsenoside Rb1 | C54H92O23 | Panax notoginseng |
| 17 | 16.26 | Ginsenoside R2 | C41H70O13 | Panax notoginseng |
| 18 | 16.44 | Ginsenoside F2 | C42H72O13 | Panax notoginseng |
| 19 | 16.48 | Ginsenoside Rh1 | C36H62O9 | Panax notoginseng |
| 20 | 16.68 | Ginsenoside Rd | C48H82O18 | Panax notoginseng |
| 21 | 16.87 | Ginsenoside Re | C48H82O18 | Panax notoginseng |
| 22 | 18.35 | Tanshindiol B | C18H16O5 | Panax notoginseng |
| 23 | 19.02 | Tanshinone IIA | C19H18O3 | Salvia miltiorrhiza |
| 24 | 20.59 | Tanshinone I | C18H12O3 | Salvia miltiorrhiza |
| 25 | 21.89 | Tanshinone IIB | C19H18O4 | Salvia miltiorrhiza |


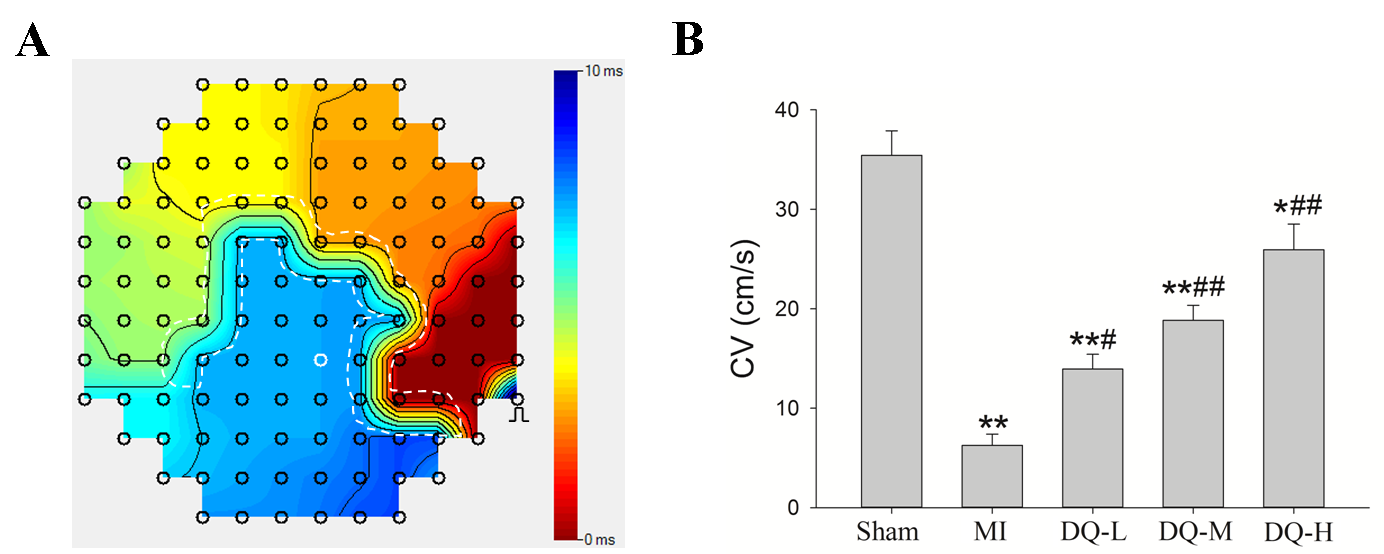


**FIGURE S2.** Effect of DQ on conduction function in post-MI rats. **A**, A representative isochronous map in the MI group. Areas of isochronal crowding were found in the MI group. The black pulse symbol indicates the site of the stimulus electrode. The white dashed lines indicate the areas of conduction block. **B**, Conduction velocities in the four groups. DQ improves the conduction velocity (*n*=5 rats/group). ***P* < 0.01, **P*< 0.05 versus sham group; ^#^*P*< 0.05, ^##^*P*< 0.01 versus MI group.
